# Caspase-11-Gasdermin D-Mediated Pyroptosis Is Involved in the Pathogenesis of Atherosclerosis

**DOI:** 10.3389/fphar.2021.657486

**Published:** 2021-04-26

**Authors:** Mengqing Jiang, Xuejing Sun, Suzhen Liu, Yan Tang, Yunming Shi, Yuanyuan Bai, Yujie Wang, Qiong Yang, Qize Yang, Weihong Jiang, Hong Yuan, Qixia Jiang, Jingjing Cai

**Affiliations:** ^1^ Department of Cardiology, The Third Xiangya Hospital, Central South University, Changsha, China; ^2^ Suzhou Science and Technology Town Foreign Language School, Jiangsu, China; ^3^ The Center of Clinical Pharmacology, The Third Xiangya Hospital, Central South University, Changsha, China; ^4^ Department of Cardiology, Tongren Hospital, Shanghai Jiao Tong University School of Medicine, Shanghai, China

**Keywords:** atherosclerosis, inflammation, pyroptosis, macrophage, caspase-11, gasdermin D, cardiovascular diseases, immunity

## Abstract

**Background:** Pyroptosis is a form of cell death triggered by proinflammatory signals. Recent studies have reported that oxidized phospholipids function as caspase-11 agonists to induce noncanonical inflammasome activation in immune cells. As the levels of oxidized phospholipids derived from ox-LDL are largely elevated in atherosclerotic lesions, this study sought to determine whether oxidized lipids trigger pyroptosis and subsequent inflammation in the pathogenesis of atherosclerosis.

**Methods and Results:** In our current study, after integrating transcriptomic data available from the Gene Expression Omnibus with data from hyperlipidemic mice and ox-LDL-treated peritoneal macrophages, we discovered that caspase-4/11-gasdermin D-associated inflammatory signaling was significantly activated. Consistently, the mRNA expression of caspase-4 and gasdermin D was upregulated in peripheral blood mononuclear cells from patients with coronary heart disease. In particular, the expression of caspase-4 was closely associated with the severity of lesions in the coronary arteries. An *in vivo* study showed that caspase-11-gasdermin D activation occurred in response to a high-fat/high-cholesterol (HFHC) diet in ApoE^−/−^ mice, while caspase-11 deletion largely attenuated the volume and macrophage infiltration of atherosclerotic lesions. An *in vitro* mechanistic study showed that caspase-11-mediated inflammation occurred partly via gasdermin D-mediated pyroptosis in macrophages. Suppressing gasdermin D in HFHC-fed ApoE^−/−^ mice via delivery of an adeno-associated virus markedly decreased lesion volume and infiltrating macrophage numbers.

**Conclusion:** Caspase-11-gasdermin D-mediated pyroptosis and the subsequent proinflammatory response in macrophages are involved in the pathogenesis of atherosclerosis. Therefore, targeting the caspase 11-gasdermin D may serve as an alternative strategy for the treatment of atherosclerosis.

## Introduction

Pyroptosis is a highly regulated cell death process and plays a pivotal role in the pathogenesis of various cardiovascular diseases (CVDs), which can be facilitated by many factors, including lipid stimulation (Libby et al., 2019; Zhaolin et al., 2019). Atherosclerosis is a CVD with low-grade chronic inflammation. Anti-lipoprotein therapies, anti-inflammatory therapies, and immunomodulatory therapies for atherosclerosis have previously been tested or are currently being tested in clinical trials (Zhao and Mallat, 2019). However, a significant number of patients continue to suffer from atherosclerosis (Bruikman et al., 2017). Therefore, the development and rigorous testing of novel targets and therapeutics that address unmet needs are urgently needed. The discovery of pyroptosis has broadened our understanding of CVDs, and targeting this process may provide new avenues for the treatment and management of atherosclerosis.

Caspase-11, a cytosolic endotoxin [lipopolysaccharide (LPS)] receptor, mediates pyroptosis under conditions of LPS stimulation in endotoxemia and bacterial sepsis (Deng et al., 2018). Human caspase-4/5 is homologous to mouse caspase-11, which has been confirmed to directly senses intracellular LPS derived from Gram-negative bacteria during macrophage inflammatory responses (Shi et al., 2014). Recognition of intracellular LPS facilitates the rapid oligomerization of caspase-11/4/5, which results in the cleavage of the pore-forming protein gasdermin D (GSDMD), cell pyroptosis, and the release of proinflammatory cytokines, e.g., IL-1β and IL-18 (Eren et al., 2020; Karmakar et al., 2020).

The accumulation of plasma cholesterol, low-density lipoprotein (LDL) cholesterol, and apolipoproteins is regarded as the main cause of the initiation and progression of clinical atherosclerosis (Back et al., 2019). Oxidized phospholipid-stimulated macrophages and dendritic cells can induce the activation of various inflammasomes (Westerterp et al., 2017). Moreover, studies have reported that oxidized phospholipids also induce noncanonical inflammasome activation via caspase-11 in immune cells (Shi et al., 2014). Additionally, the structures of lipids or fatty acids (i.e., ox-PAPC10 and stearoyl lysophosphatidylcholine 11) are similar to that of lipid A, which can bind to caspase-11 and subsequently induce caspase-11-dependent interleukin (IL)-1 release (Zanoni et al., 2016). These findings inspired us to explore whether oxidized lipids trigger pyroptosis and subsequent inflammation in the pathogenesis of atherosclerosis. Understanding the mechanism of pyroptosis in atherosclerosis could facilitate the discovery of potential therapeutic targets that may be beneficial for atherosclerosis treatment.

## Methods

### Microarray Data Retrieval and Analysis

To identify differences in gene expression between models fed a normal chow diet or a high-fat high-cholesterol (HFHC) diet, we retrieved four mouse microarray datasets (GSE2372, GSE39264, GSE40156, and GSE60086) from the National Center for Biotechnology Information Gene Expression Omnibus (GEO) website (https://www.ncbi.nlm.nih.gov/geo/) (Houde and Van Eck, 2018). We downloaded robust multiarray-averaging normalized microarray expression profiles and the corresponding annotation files. Gene probe identification was matched to the corresponding gene symbol after the files were downloaded. For situations in which multiple probes were used for one gene, we retained the probe that showed a significant gene expression value (adjusted *p*-value) after deleting the non-mRNA probes. The identification of significant differentially expressed genes (DEGs) was performed according to the matched matrix file. The GSE2372 dataset, tested on the GPL1261 platform based on the Affymetrix Mouse Genome 430 2.0 Array (*Mus musculus*), and the GSE39264 dataset, tested on the GPL8759 platform based on the Affymetrix HT Mouse Genome 430A Array, contained gene expression data that was used determine gene expression changes in the aorta during atherosclerotic lesion progression. The GSE40156 dataset, tested on the GPL1261 platform based on the Affymetrix Mouse Genome 430 2.0 Array, and the GSE60086 dataset, tested on the GPL213730 platform based on the Affymetrix Mouse Gene 1.0 ST Array, contained gene expression data used to examine the hyperlipidemia-induced modulation of vascular cell gene expression during early atherosclerosis. The limma package (version 3.30.3) was used to identify DEGs between the aorta of HFHC-treated mice and that of control mice. The q-value between gene expression levels was determined using the T-test and adjusted using the Benjamin-Hochberg (BH) method. Genes that met the cutoff criteria of a q-value < 0.05 and |log2 (fold change (FC)| > 1.5 were considered DEGs. Gene Ontology (GO) term (Lopategi et al., 2019) and Kyoto Encyclopedia of Genes and Genomes (KEGG) (Schroder et al., 2010) pathway enrichment analyses of DEGs were performed using the DAVID bioinformatic database (https://david.ncifcrf.gov/, version 6.8) (Grebe et al., 2018) with thresholds of count ≥2 and *p*-value < 0.05.

### RNA Sequencing

Total RNA was isolated from ox-LDL-treated peritoneal macrophages using TRIzol. Then, RNA degradation and contamination were examined with 1% agarose gels. We checked RNA purity by using a NanoPhotometer spectrophotometer (IMPLEN, Munich, Germany), and RNA concentration was measured using the Qubit RNA Assay Kit with a Qubit 2.0 fluorometer (Life Technologies, Carlsbad, CA, United States). RNA sequencing was performed with a standard protocol using a BGISEQ-500 platform (BGI, Guangdong, China). Data quality was assessed with FastQC, and reads were aligned to a mouse genome (mm9) with HiSat2 2.0.5, sorted with SamTools 1.4 and assembled with StringTie (1.3.3.13 R package); scater was used for quality control. After log-normalizing the data, the principal component analysis was performed for dimension reduction. Heatmaps and volcano plots of the RNA-seq data were plotted with the R package ggplot2 (version 3.1.0). GO, KEGG, and genome pathway analyses were performed with DAVID, as previously described. Gene set enrichment analysis (GSEA) was performed with GSEA (version 3.0) software.

### Human Samples

Human peripheral blood samples were collected from patients with coronary arterial disease (*n* = 28) upon hospital admission at the Third Xiangya Hospital of Central South University. Only nonacute coronary arteriopathy patients diagnosed by coronary arteriography were enrolled in our study. Patients meeting any of the following criteria were excluded from the study: 1) acute myocardial infarction; 2) acute infection; 3) diabetes; 4) malignant tumor; or 5) liver and kidney dysfunction. The severity of coronary arterial disease was defined by the SYNTAX score. The control group included coronary angiography patients with no stenosis in the coronary arteries. Clinical characteristics of the included patients are shown in Supplementary Table S1. All procedures that involved human sample collection were approved by the ethics committee of the Third Xiangya Hospital of Central South University and adhered to the principles of the Declaration of Helsinki.

### Animals

ApoE^−/−^ mice were purchased from the Model Animal Research Center of Nanjing University (Nanjing, China). Caspase-11^−/−^ mice were from Professor Lu Ben, Third Xiangya Hospital of Central South University. All mice were on the C57BL/6 background, and ApoE^−/−^Caspase-11^−/−^ mice were obtained by backcrossing ApoE^−/−^ mice to caspase-11^−/−^ mice for more than 10 generations. The genotypic identification of various strains is shown in Supplementary Figure S1 and Supplementary Table S2. All animals were maintained under a 12-h light/dark cycle in a pathogen-free facility at the animal facility of Central South University (Changsha, Hunan, China) and Hunan Normal University (Changsha, Hunan, China) at 22 ± 2°C, and the humidity was maintained at 50–60%. All animal protocols were approved by the Animal Care and Use Committee of the Third Xiangya Hospital of Central South University and Hunan Normal University.

### Establishment of an Atherosclerotic Mouse Model

An atherosclerotic model was established (shown in Supplementary Figure S2) with 8- to 10-week-old male ApoE^−/−^ mice fed a HFHC diet (protein, 20%; fat, 40%; carbohydrates, 40%; cholesterol, 1.25%) (D12108C, Research Diets, New Brunswick, NJ, United States) for twelve continuous weeks. ApoE^−/−^ mice fed normal chow served as controls.

### Adeno-Associated Virus-5 Construction and Injection

An AAV-5 delivery system was used to suppress gasdermin D expression in ApoE^−/−^ mice. The AAV-5 delivery system carried a gasdermin D-specific construct to suppress gasdermin D expression in ApoE^−/−^ mice and was developed by Hanbi (Shanghai, China) (Jain and Ridker, 2005). The titers of the vector genome were measured with qPCR performed with vector-specific primers (shown in Supplementary Table S3). APOE^−/−^ mice were injected via the tail vein with 100 μl of virus containing 2 × 10^8^ μg of AAV-5 vector genome at 8 weeks of age.

### Flow Cytometry Analysis of Macrophages Within the Mouse Aorta

Mouse aorta macrophages were isolated as previously described (Sabatine et al., 2017). Briefly, aortic arteries were dissected from the aortic arch to femoral bifurcations and digested in an enzyme solution containing papain (2 mg/ml) (P4762, Sigma-Aldrich, Santa Clara, CA, United States) in phosphate-buffered saline (PBS) at 37°C for 1 h. A single-cell suspension was prepared by passing the aortic pieces through a strainer and subsequently stained for flow cytometry. Cell suspensions were stained with (APC)-Cy7-conjugated anti-Zombie (423101, BioLegend, San Diego, CA, United States), V500-conjugated anti-CD45 (103138, BioLegend, San Diego, CA, United States), PE-Cy7-conjugated anti-CD11b (123109, BioLegend, San Diego, CA, United States), and PE-conjugated anti-F4/80 (123109, BioLegend, San Diego, CA, United States) antibodies for 15 min at 4°C. Absolute cell counts were detected by flow cytometry (BD FACSCalibur, United States) and analyzed using FlowJo software version X (Tree Star Inc., United States).

### Isolation of Human Peripheral Blood Monocytes

Whole blood samples from patients with coronary arterial disease or control patients with a coronary angiography SYNTAX score of zero were processed within 2 h of sample collection. The whole EDTA k+-anticoagulated blood sample was diluted 1:1 with PBS (SH30256.01, HyClone, UT, United States). Then, 4 ml of diluted blood was added to 3 ml of Ficoll-Paque Plus (17144003, GE Healthcare Life Sciences, Pittsburgh, PA, United States) and centrifuged for 40 min at 400 g and 18–22°C. After centrifugation, the peripheral blood mononuclear cell (PBMC) layer was collected and washed twice in PBS at 4°C, and total monocytes were isolated from the human PBMCs using the MojoSort™ Human Pan Monocyte Isolation Kit (480060, BioLegend, San Diego, CA, United States) according to the instructions.

### Isolation of Mouse Peritoneal Macrophages

Mouse peritoneal macrophages were isolated and cultured as previously described (Lehrer-Graiwer et al., 2015). Briefly, 8- to 10-week-old mice were intraperitoneally injected with 3 ml of sterile 3% thioglycollate (70157, Sigma-Aldrich, Santa Clara, CA, United States) broth to elicit peritoneal macrophages. Peritoneal macrophages were collected by three rounds of peritoneal cavity lavage using 5 ml of RPMI 1640 medium (SH30809.01, Gibco, Carlsbad, CA, United States) on the third day after thioglycollate infusion. Peritoneal macrophages collected from the peritoneal cavity were resuspended in RPMI 1640 medium supplemented with 10% fetal bovine serum (10099141, Gibco, Carlsbad, CA, United States) and 1% antibiotics-antimycotic containing 10,000 units/ml penicillin, 10,000 μg/ml streptomycin, and 25 μg/ml Fungizone™ (15240-062, Gibco, Carlsbad, CA, United States). Peritoneal macrophages (10^6^ cells per well) were plated in 12-well plates for further treatment.

### Supernatant Cytokine Parameter Analysis

The concentration of the cytokine IL-1β (VAL601, Novus, San Diego, CA, United States) and IL-18 (DY7625-05, Novus, San Diego, CA, United States) in the supernatant was measured by ELISA according to the manufacturer’s instructions. Briefly, peritoneal macrophage supernatant was gathered and centrifuging at 400g for 5 min, following add 100 μl of diluted sample to each well in a 96-well plate according to the manufacturer’s instructions, then the optical density (OD value) of each well were detected by microplate reader (Perkin Elmer, Waltham, Mass, United States) at 450 nm wavelength and the level of IL-1β and IL-18 were determined using Microsoft's excel software (Tree Star Inc., United States) based on the standard curve according to the manufacturer’s instructions. LDH release was detected using an LDH assay kit (C0017, Beyotime Biotechnology, Shanghai, China) according to the manufacturer’s instructions. LDH release (%) = (absorbance of samples − absorbance of the blank hole)/(absorbance of Maximum enzyme activity − absorbance of the blank hole) × 100.

### Hematoxylin and Eosin, Oil Red O and Immunofluorescence Staining

To visualize lipid accumulation in the aorta, we performed H&E (GHS216, 48900, Sigma-Aldrich, Santa Clara, CA, United States) and ORO staining (O0625, Sigma-Aldrich, Santa Clara, CA, United States) on frozen sections of 4% paraformaldehyde-fixed aortic roots. To identify macrophage infiltration in atherosclerotic lesions, immunofluorescence staining for macrophages was performed using an anti-F4/80 antibody (ab6640, Abcam, Cambridge, MA, United States). The local expression of caspase-11 and gasdermin D was determined by immunofluorescence staining using anti-caspase-11 (919916, R&D Systems, MN, United States) and anti-gasdermin D (SC-393581, Santa Cruz, Dallas, TX, United States) antibodies.

Fluorescence images were captured by fluorescence microscopy (Perkin Elmer, Waltham, Mass, United States), and lesion size was quantified using ImageJ software (National Institutes of Health, United States). Immunofluorescence images were captured and recorded by a fluorescence microscope (ZEISS) with Vert.A.1 software, and the signal intensity of target proteins was calculated by ImageJ software (National Institutes of Health, United States).

OCT-embedded tissues were sectioned (10 µm) and subjected to H&E and ORO staining as previously reported. Histological staining images were observed and recorded using a light microscope (PerkinElmer). Signal intensity was determined by ImageJ software (National Institutes of Health, United States). The infiltration of inflammatory cells into aorta sections was tested by immunofluorescence staining with primary antibodies against F4/80 (ab6640, Abcam, Cambridge, MA, United States), caspase-11 (919916, R&D, MN, United States), IL-1β (AF5103, Affinity, Cincinnati, OH, United States), IL-18 (DF6252, Affinity, Cincinnati, OH, United States), and gasdermin D (393581, Santa Cruz, Dallas, TX, United States). After incubation with a primary antibody overnight at 4°C, aorta sections were incubated with a secondary antibody, washed and then mounted with DAPI (236276, Roche, Switzerland). Immunofluorescence images were captured and recorded by a fluorescence microscope (ZEISS) with Vert.A.1 software.

### Quantitative Real-Time PCR and Western Blot Analyses

RT-PCR and western blot analyses were conducted as previously described with minor modifications (O'Donoghue et al., 2014). For the RT-PCR assay, total mRNA was extracted using TRIzol reagent (15596-026, Invitrogen, Carlsbad, CA, United States) and then reverse transcribed into cDNA. PCR amplification was performed with SYBR Green PCR Master Mix (1725214, Bio-Rad, Hercules, CA, United States). The mRNA expression of genes of interest was normalized to that of Gapdh. The primer pairs used in this study are listed in Supplementary Table S4.

For western blot analysis, total protein samples were extracted from murine aortic tissue and peritoneal macrophages using RIPA lysis buffer (P0013BB, Beyotime, China). Protein concentrations were examined using the Pierce^®^ BCA Protein Assay Kit (P0012, Beyotime, China). The obtained protein (40 µg) was separated on a 10% SDS-PAGE gel, transferred to a polyvinylidene fluoride (PVDF) membrane (IPVH0010, Merck Millipore, Darmstadt, Germany), blocked with 5% milk which is dissolved in TBST (50 mM Tris-HCl, pH 7.5, 150 mM NaCl, 0.1% Tween 20) for 1 h, and incubated with a primary antibody at 4°C for 12 h and a secondary antibody at room temperature for 1 h, and the relevant signal intensity was determined using ImageJ software (National Institutes of Health, United States). β-Actin served as a loading control.

### Correlation Coefficient Analysis

In this study, the Pearson correlation coefficient analysis was applied to describe the proportion of the variance in the dependent variable that is predictable from the independent variables. Specifically, the association of the mRNA levels of caspase-4 in PBMCs with the SYNTAX score, which indicated the severity of coronary atherosclerosis was analyzed. The correlation coefficient R^2^ measures the strength and direction of a linear relationship between two analyzed variables on a scatterplot. *p*-value less than 0.05 defined as statistically significant.

### Statistical Analysis

All data are expressed as the mean ± standard deviation (SD). Data were first analyzed for normality test (by Shapiro-Wilk test or D’Agostino and Pearson test where indicated). Data passed the normality test were then assessed by unpaired two-tailed t test (two groups) or one-way ANOVA with Tukey’s test (three groups or more), where indicated. Non-normally distributed data (two groups) were analyzed by Mann-Whitney test. *p* < 0.05 was considered to be statistically significant. For linear regression analysis, data were also first tested and passed the normality test (D’Agostino and Pearson test). No experiment-wide multiple test correction was applied in our study. Representative images were chosen for similarity to the quantification data (close to the average expression) and the image quality. Statistical calculations in the animal experiments were performed on at least three independent experiments. A *p*-value less than 0.05 was considered to indicate statistical significance. Statistical analysis was performed using the statistical software GraphPad Prism 7 (www.graphpad.com/scientific-software/prism/).

## Results

### Caspase-11-Gasdermin D-Mediated Pyroptosis Signaling Was Involved in the Pathogenesis of Atherosclerosis

**FIGURE 1:**
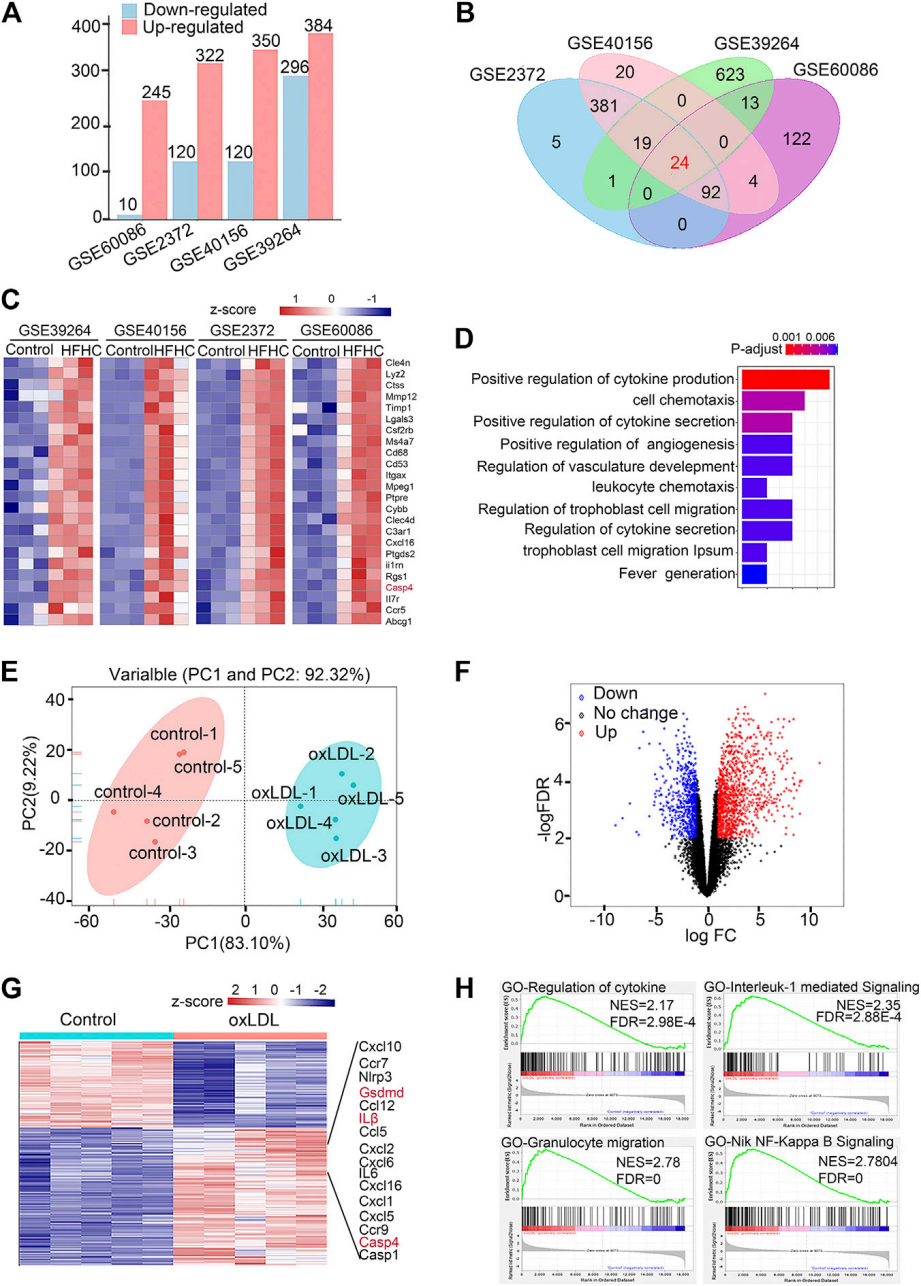
Caspase-11-gasdermin D medicated pyroptosis signaling is involved in the pathogenesis of atherosclerosis. **(A)** Histogram showing numbers of different expression genes (DEGs) of included datasets in GEO database. DEGs with the cut-off criteria of −log10 False Discovery Rate (−logFDR) > 2 and |log2 fold change (FC)| > 2 were colored in red for up-regulated gens while in blue for down-regulated genes. **(B)** Venn diagrams depict 24 genes with consistent pattern of expression across four available transcriptomic datasets from aortic arterial tissue from C57BL/6J or ApoE^−/−^ mice fed with normal chow diet or HFHC in the GEO database. **(C)** Heatmap of 24 DEGs in four datasets of mouse aortas from normal chow diet or HFHC diet. The abscisa represents different GEO datasets and samples, and the ordinate represents different genes. The red boxes indicate up-regulated genes, and the blue boxes indicate down-regulated genes. **(D)** GO term enrichment analysis illustrating number of significantly up-regulated in the most affected up-regulated genes common in HFHC diet mice aortas compared to the controls. **(E)** Principal component analysis of bulk RNA-seq data in peritoneal macrophages primed with LPS followed by 16 h of ox-LDL stimulation of control and LDL-treated macrophages. **(F)** Volcano plot indicating transcriptomic changes in control and LDL-treated macrophages. The red dots indicate up-regulated genes, and the blue dots indicate down-regulated genes. **(G)** Heatmap with differentially expressed genes in control and LDL-treated macrophages. DEGs with the cut-off criteria of −log10 False Discovery Rate (−logFDR) > 2 and |log2 fold change (FC)| > 2 were colored in red for up-regulated gens while in blue for down-regulated genes. The abscisa represents samples, and the ordinate represents different genes. The red boxes indicate up-regulated genes, and the blue boxes indicate down-regulated genes. **(H)** Gene Set Enrichment Analysis in control and LDL-treated macrophages. GEO, Gene Expression Omnibus; HFHC, high fat high cholesterol; GO term, Gene Ontology term; PCA, Principal component analysis.

Since previous studies demonstrated that oxidized phospholipids function as caspase-11 agonists to induce noncanonical inflammasome activation in immune cells and the levels of oxidized phospholipids derived from ox-LDL are largely elevated in atherosclerotic lesions, we integrated the transcriptomic data available from the GEO database with data from hyperlipidemic mice and ox-LDL-treated peritoneal macrophages (details are shown in Supplementary Table S5). The upregulated and downregulated genes in the control and overnutrition diet-challenged groups were identified by analyzing DEGs (Figure 1A). The Venn diagram shows 24 genes with consistent expression patterns across the four DEG datasets (Figure 1B). Notably, 24 genes at the intersection exhibited upregulated expression in aortic tissue from mice fed the overnutrition diet (Figure 1C), and caspase-4/11 was among the top upregulated genes across the datasets. According to GO analysis, the top three gene-enriched biological processes were related to cytokine production and cell chemotaxis (Figure 1D). These results indicate that the proinflammatory response plays a dominant role in the pathogenesis of atherosclerosis. Since lesion macrophages are essential in mediating the proinflammatory response, we further verified whether the caspase-4/11-associated inflammatory response is triggered in ox-LDL-treated macrophages. Bulk RNA-seq was performed to assess peritoneal macrophages primed with LPS and then stimulated with ox-LDL for 16 h. The principal component analysis gene expression profiles of the control and ox-LDL-treated groups were clearly distinct (Figure 1E). Principal component loading plots 1 (PC1) and 2 (PC2) described 92.32% of the total variation in the control and ox-LDL treated groups. Among the DEGs that met the cutoff criteria of a −log10 (false discovery rate (FDR)) > 2 and |log2 (fold change (FC)| > 2, the upregulated genes are shown in red, and the downregulated genes are shown in blue. A total of 1,388 downregulated and 855 upregulated genes were identified (Figures 1F,G). GSEA showed that the dominant upregulated pathways in ox-LDL-treated macrophages were associated with the IL-1-mediated signaling pathway, response to cytokine stimulus, and granulocyte migration (Figure 1H). Genes involved in the caspase-11-gasdermin D pathway and the downstream signaling pathway were among the top 16 upregulated genes in macrophages treated with ox-LDL (Figure 1G). The evidence from public databases and ox-LDL-treated macrophages indicate that caspase-4/11-gasdermin D-associated inflammation may be involved in the progression of atherosclerosis.

### Caspase-4/11 Was Activated in Atherosclerotic Arteries and Ox-LDL-Treated Macrophage Pyroptosis

**FIGURE 2:**
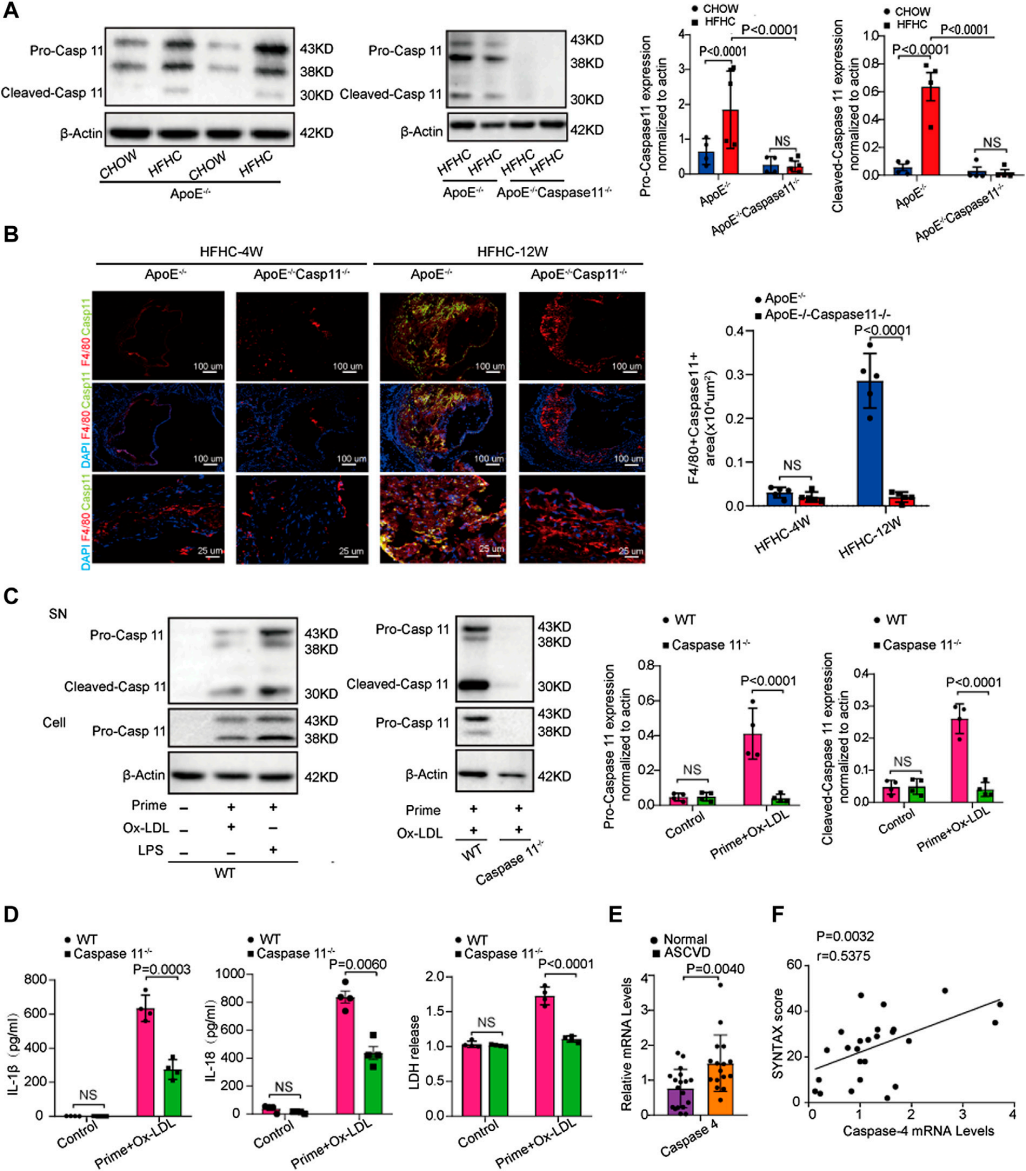
Caspase-4/11 accelerates lipid-promoted atherosclerosis and ox-LDL-treated macrophage pyroptosis. **(A)** Left, western blot analysis and quantification of caspase-11 and its cleaved fragment expression in aortic arteries from WT, ApoE^−/−^, and ApoE^−/−^Caspase-11^−/−^ mice fed with chow diet and HFHC diet for twelve weeks (*n* = 4 per group). Middle, quantification results of the level of cleaved caspase-11 in indicated groups. Right, quantification results of the level of pro-caspase-11 in indicated groups. **(B)** Left, representative images of caspase-11 (green), F4/80 (red) and DAPI (blue) in immunofluorescent staining of aortic root sections of indicated group, and quantification of the F4/80 and caspase-11 positive area of aortic root sections (*n* = 4 per group). Right, quantification results of the level of F4/80 and caspase-11 positive area of aortic root sections in the indicated groups. **(C)** Left, representative immunoblots of cleaved caspase-11 in peritoneal macrophage primed with 16-h ox-LDL stimulation in the indicated groups (*n* = 4 per group). Right, quantification results of the level of cleaved caspase-11 in indicated groups. **(D)** IL-1β (Left), IL-18 (Middle) and LDH release (Right) level secreted in peritoneal macrophage primed with LPS for 5 h and then treated with ox-LDL for 16 h after LPS administration was measured via enzyme-linked immunosorbent assay (ELISA) kits (*n* = 4 in each group). **(E)** Quantification results of the mRNA levels of caspase-4 genes in peripheral blood monocytes in patients with coronary heart diseases and the control group (*n* = 10 in each group). **(F)** Pearson comparison analyses of the correlations between caspase-4 mRNA levels in PBMC and SYNTAX score of patients with coronary heart diseases (*n* = 25). *p* < 0.05 for all of these correlations, by Pearson's rank correlation coefficient analysis. Data shown are mean ± SD **(A–F)**. Data were first analyzed and passed normality test [Shapiro-Wilk test in **(A–E)**, D’Agostino and Pearson test in **(F)**]. *p* values were shown and assessed by one-way ANOVA with Tukey’s test **(A, B)**, by Mann-Whitney test **(C)** and by unpaired two-tailed *t* test **(D, E)**. All of the *p* values were labeled on the pictures and *p* < 0.05 was considered to indicate statistical significance. Ox-LDL, Oxidized low-density lipoprotein; PBMC, peripheral blood monoculear cell; SYNTAX, Synergy Between Percutaneous Coronary Intervention with Taxus and Cardiac Surgery.

We further tested whether caspase-11 expression is upregulated in an atherosclerotic animal model. Western blot analysis showed that caspase-11 was activated in ApoE^−/−^ mice after twelve weeks of HFHC diet feeding. The level of cleaved caspase-11 at 30kD was significantly upregulated in ApoE^−/−^ mice fed a HFHC diet compared to ApoE^−/−^ mice fed a chow diet. Interestingly, the levels of pro-caspase-11 at 38kD and 43kD were upregulated in ApoE^−/−^ mice fed a chow diet and wild-type (WT) mice fed a HFHC diet (Figure 2A). Immunofluorescence staining revealed that the expression of caspase-11 was upregulated in macrophages resided in the atherosclerotic plaques in ApoE^−/−^ mice after 12-week of HFHC diet. Consistently, the expression of inflammatory cytokines (e.g., IL-1β and IL-18) associated with cell pyroptosis were increased in the section of atherosclerotic plaques in HFHC treated ApoE^−/−^ mice. The induction of cytokines was suppressed in caspase-11^−/−^ApoE^−/−^ mice with 12-week of HFHC diet. The image also showed that HFHC treatment fails to induce a significant increase in the expression of caspase 11 and inflammatory cytokines at 4-week time point in ApoE^−/−^ strain (Supplementary Figure S3). The ratio of apoptotic cell and the release of proinflammatory cytokines (e.g., IL-1β, IL-18) were increased after ox-LDL exposure in ApoE^−/−^ mice derived peritoneal macrophage (Figures 2C,D), while ox-LDL induced cell apoptosis and cytokine release were significantly decreased in ApoE^−/−^Caspase-11^−/−^ mice isolated peritoneal macrophage (Supplementary Figure S4). As circulating monocytes have been shown to be involved in the development of atherosclerotic lesions by invading the intima and undergoing transformation into foam cells, we further tested whether the expression of caspase-4, the gene in humans homologous to mouse caspase-11, is altered in circulating monocytes in patients with coronary angiogram (CAG)- confirmed coronary heart disease (CHD). We found that the mRNA expression of caspase-4 in PBMCs was higher in patients with CHD than in normal controls (Figure 2E). The level of caspase-4 mRNA expression in PBMCs was positively correlated with the severity of coronary atherosclerosis, which was indicated by the SYNTAX score (Figure 2F). These data suggest that caspase-4/11 and its role in macrophages may be pivotal in the pathogenesis of atherosclerosis.

### Genetic Deletion of Caspase-11 Attenuated Atherosclerotic Plaque Progression and Macrophage Infiltration in ApoE^−/−^ Mice

**FIGURE 3:**
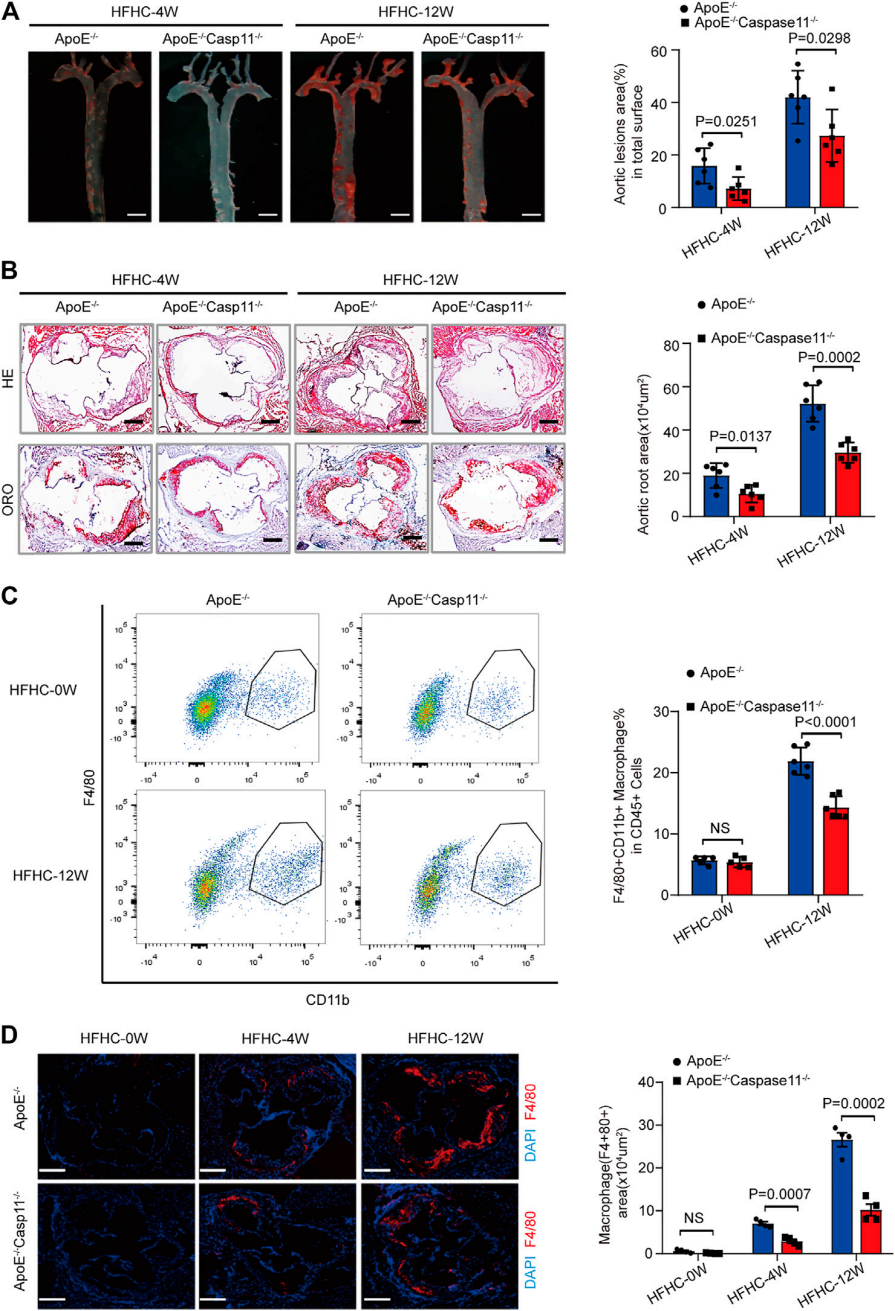
Genetic deletion of caspase-11 attenuated atherosclerosis plaque progression and macrophage infiltration in ApoE^−/−^ mice. **(A)** Left, overall comparison of representative whole-aortas stained with Oil-Red-O staining (*n* = 5 mice per group; scale bar, 1 mm). Right, quantitative results of the stained area in the entire aortas in ApoE^−/−^ and ApoE^−/−^Caspase-11^−/−^ mice with 4 or 12-week HFHC diet. **(B)** Left, histological analysis of aortic root sections stained with H&E and ORO staining in the aortic root sections in ApoE^−/−^ and ApoE^−/−^Caspase-11^−/−^ mice after 4 or 12-week HFHC diet (*n* = 5 mice per group; scale bar, 100 μm). Right, quantitative results for the lesion formation in the aortic root sections in the indicated groups (*n* ≥ 20 fields per group). **(C)** Left, flow cytometric analysis of aortic macrophage content, determined as F4/80+CD11b+ cells per aorta and statistical results for aortic macrophage content in ApoE^−/−^ and ApoE^−/−^Caspase-11^−/−^ mice after 0 and 12-week HFHC diet treatment (*n* = 5 mice per group). Right, quantitative results of flow cytometric analysis. **(D)** Left, representative images of F4/80 (red) and DAPI (blue) in aortic root sections in ApoE^−/−^, ApoE^−/−^Caspase-11^−/−^ mice after 0, 4 and 12-week of HFHC treatment (*n* = 5 mice per group; scale bar, 100 μm). Right, quantification analysis of the F4/80 positive area of aortic root sections in the indicated groups (*n* ≥ 20 fields per group). Data shown are mean ± SD **(A–D)**. Data were first analyzed and passed normality test (Shapiro-Wilk test). *p* values were shown and assessed by Mann-Whitney test **(A–D)**. All of the *p* values were labeled on the pictures and *p* < 0.05 was considered to indicate statistical significance. ORO, Oil-Red-O; H&E, hematoxylin and eosin; HFHC, High fat and high cholesterol.

To further verify whether caspase-11 depletion can slow the development of atherosclerosis and the subsequent inflammatory response, we generated ApoE^−/−^Caspase-11^−/−^ mice that were fed a HFHC diet or normal chow for 4 or 12 weeks. The baseline characteristics of the aorta were comparable among the various groups of mice. ORO staining revealed that the numbers of atherosclerotic lesions were markedly reduced in ApoE^−/−^Caspase-11^−/−^ mice compared with ApoE^−/−^ mice fed a HFHC diet for 4 or 12 weeks (Figure 3A). Consistently, the incidence of atherosclerotic lesions at the aortic root was also significantly reduced in ApoE^−/−^Caspase-11^−/−^ mice after 4 or 12 weeks of HFHC diet feeding, which was shown by H&E and ORO staining (Figure 3B). To further determine whether caspase-11 depletion protects against atherosclerosis via modulation of macrophage-mediated vascular inflammation and immune infiltration, we performed flow cytometry to analyze macrophages in aortic arteries. The numbers of CD11b+F4/80+ macrophages in aortic arteries were increased after 12 weeks of HFHC diet feeding, whereas caspase-11 deletion attenuated macrophage infiltration into aortic arteries (Figure 3C). Similar results were obtained by immunofluorescence staining of the aortic root, and the macrophage infiltration area (marked by F4/80) in the aortic root was reduced in ApoE^−/−^Caspase-11^−/−^ mice fed a HFHC diet (Figure 3D). Furthermore, immunofluorescence staining also revealed that the increased numbers of macrophages in the aortic root were mainly confined to proliferative atherosclerotic plaques and progressively increased with the extension of the HFHC diet feeding time (Figure 3D). These data suggested that the genetic deletion of caspase-11 attenuated atherosclerotic plaque progression by reducing macrophage infiltration.

### Caspase 11 Deficiency Attenuated Atherosclerosis by Blocking the Activation of Gasdermin D in Macrophages

**FIGURE 4:**
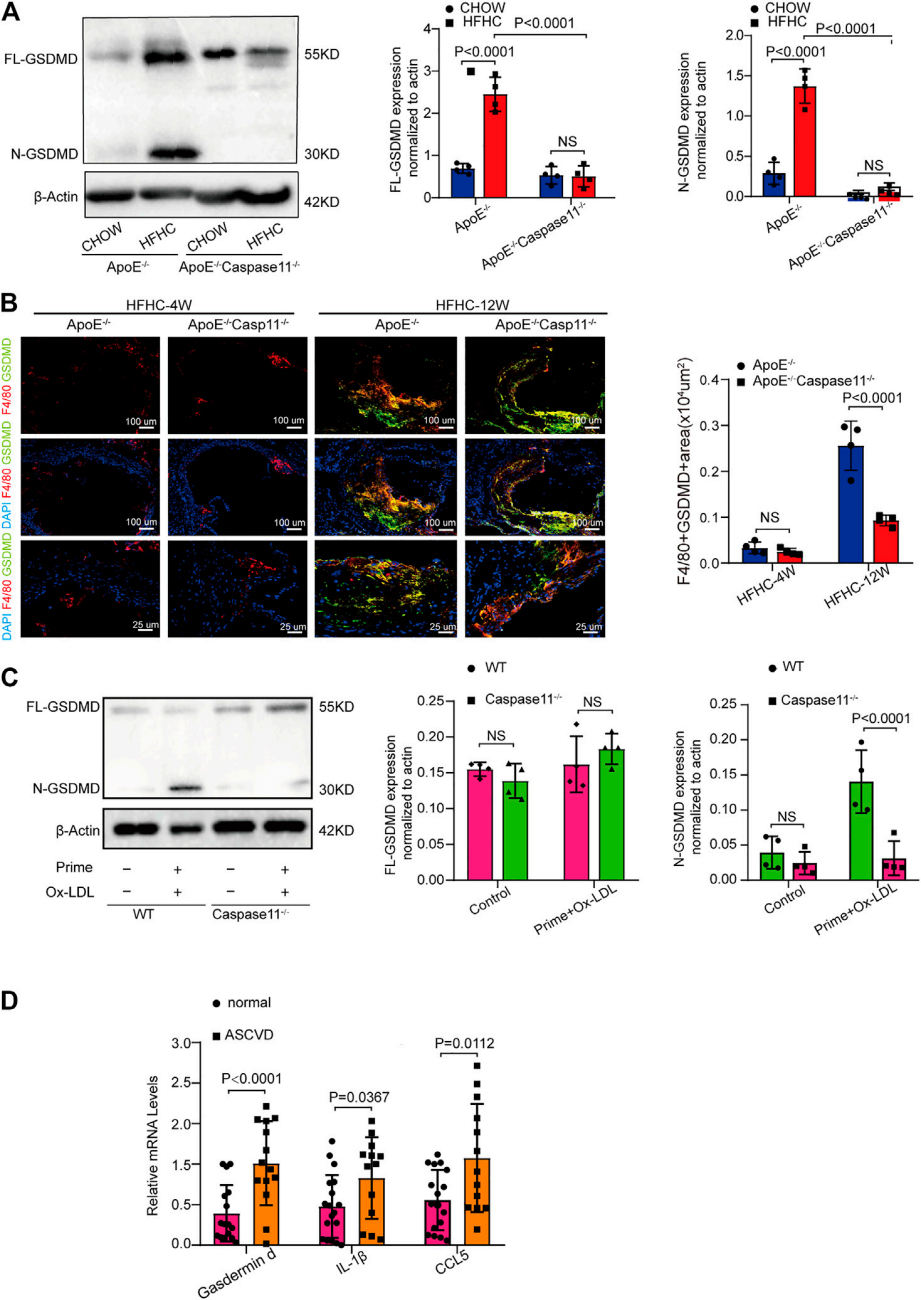
Caspase-11 deficiency attenuates atherosclerosis via blocking the activation of gasdermin D in macrophage. **(A)** Left, representative western blot of the cleaved and total protein levels of gasdermin D in the aortas of WT, ApoE^−/−^ and ApoE^−/−^Caspase-11^−/−^ mice after twelve weeks of chow diet or HFHC diet (*n* = 4 mice per group). Middle, quantitative analysis of the levels of N-GSDMD. Right, quantitative results of the protein levels of FL-GSDMD. **(B)** Left, representative images of gasdermin D (green), F4/80 (red) and DAPI (blue) of the F4/80 and gasdermin D positive area of aortic root sections in ApoE^−/−^ and ApoE^−/−^Caspase11^−/−^ mice after 4 and 12 weeks of HFHC diet (*n* = 4 per group). Right, quantification analysis of the F4/80 and gasdermin D positive area of aortic root sections in the indicated groups (*n* ≥ 20 fields per group). **(C)** Left, the protein expression of the cleaved and total protein levels of gasdermin D in peritoneal macrophage from WT and caspase-11^−/−^ mice primed with LPS for 5 h and then treated with ox-LDL for 16 h after LPS administration was determined by western blotting. Middle, quantitative analysis of the levels of N-GSDMD. Right, quantitative results of the protein levels of FL-GSDMD (*n* = 4 samples per group). **(D)** The quantification of gasdermin D, IL-1β, CCL5 mRNA levels in peripheral blood monocytes in patients with coronary heart diseases and the control group (*n* = 10 per group). Data shown are mean ± SD **(A–D)**. Data were first analyzed and passed normality test (Shapiro-Wilk test). *p* values were shown and assessed by one-way ANOVA with Tukey’s test **(A)**, by Mann-Whitney test **(B, C)** and by unpaired two-tailed *t* test (D). All of the *p* values were labelled on the pictures and *p* < 0.05 was considered to indicate statistical significance. GSDMD, gasdermin D.

Cytoplasmic gasdermin D is the key substrate for caspase-11 and can be cleaved by caspase-11 to create an N-terminal fragment that functions as a key determinant for proinflammatory cell death (Kayagaki et al., 2015). We tested whether caspase-11 deficiency attenuates atherosclerosis by blocking the activation of gasdermin D. The levels of both full-length gasdermin D (GSDMD-FL) and the pyroptosis-inducing fragment GSDMD-N were upregulated in the aorta of ApoE^−/−^ and WT mice fed a HFHC diet for 12 weeks compared with those fed a chow diet (Figure 4A). Immunofluorescence staining revealed that the upregulated gasdermin D expression was mainly confined to plaque F4/80 positive macrophages in the aortic root and that the gasdermin D staining area increased with the extension of the HFHC diet feeding time (Figure 4B). The levels of the pyroptosis-inducing fragment GSDMD-N in ApoE^−/−^ aortic tissue were reversed by depleting caspase-11 (Figure 4A). Consistently, as shown by immunofluorescence staining, gasdermin D-stained macrophage numbers were decreased in caspase-11-depleted ApoE^−/−^ mice (Figure 4B). Western blot analysis showed that gasdermin D was activated in ox-LDL-treated macrophages from WT mice, while its activation was markedly attenuated in ox-LDL-treated macrophages from caspase 11^−/−^ mice (Figure 4C). Further, we found that the mRNA expression of gasdermin D and inflammatory cytokines (represented by IL-1β and CCL5) was upregulated in PBMCs from patients with CAG-proven CHDs (Figure 4D). These results suggest that caspase-11 promotes vascular inflammation and that the development of atherosclerotic lesions is mediated by gasdermin D activation in plaque macrophages.

### Suppression of Gasdermin D Attenuated Atherosclerotic Plaque Progression and Macrophage Infiltration in ApoE^−/−^ Mice

**FIGURE 5:**
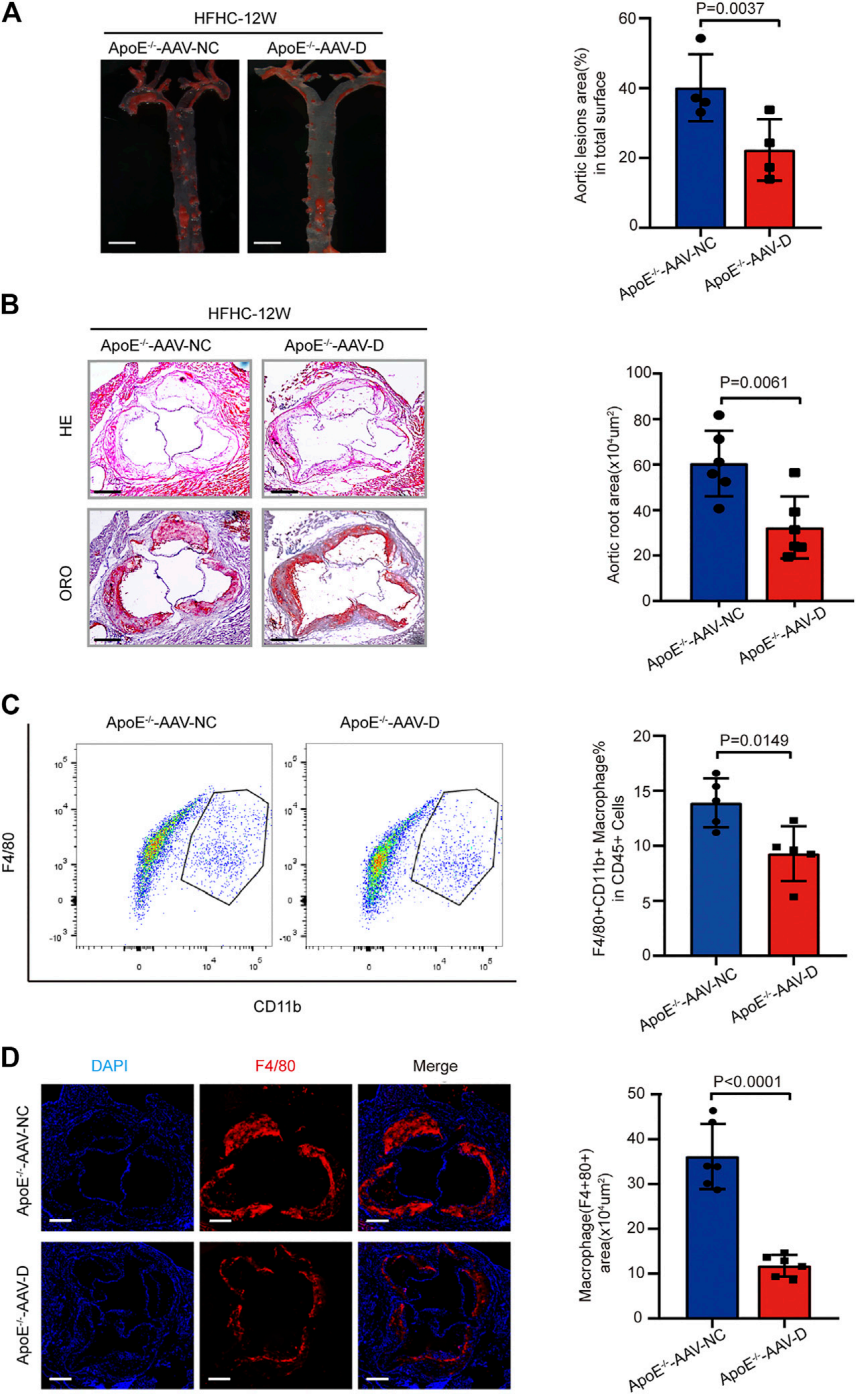
Suppression of gasdermin D attenuated atherosclerosis plaque progression and macrophage infiltration in ApoE^−/−^ mice. **(A)** Left, overall comparison of representative whole-aortas stained with Oil-Red-O staining (*n* = 5 mice per group; scale bar, 1 mm). Right, quantitative results of the stained area in the entire aortas of ApoE^−/−^ mice administered with vector AAV-gasdermin D or the control vector AAV-control after 12 weeks of HFHC diet treatment. **(B)** Left, histological analysis of aortic root sections stained with H&E and ORO staining in the aortic root sections of ApoE^−/−^ mice administered with vector AAV-GSDMD or the control vector AAV-control after 12 weeks of HFHC diet treatment (*n* = 4 mice per group; scale bar, 100 μm). Right, statistical analysis of the stained atherosclerotic lesion area in the aortic root sections by ORO staining in the indicated groups (*n* ≥ 20 fields per group). **(C)** Left, flow cytometric analysis of aortic macrophage content, determined as F4/80 and CD11b positive cells in ApoE^−/−^ mice administered vector AAV-GSDMD or the control vector AAV-control after 12 weeks of HFHC diet treatment (*n* = 4 mice per group). Right, statistical analysis of macrophage content in the indicated groups. **(D)** Left, representative images of F4/80 (red) and DAPI (blue) in aortic root sections of the F4/80 positive area of aortic root sections in ApoE^−/−^ mice administered vector AAV-GSDMD or the control vector AAV-control after 12 weeks of HFHC diet treatment (*n* = 4 mice per group; scale bar, 100 μm). Right, quantification analysis of the F4/80 positive area and aortic area ratio in the indicated groups (*n* ≥ 20 fields per group). Data shown are mean ± SD **(A–D)**. Data were first analyzed and passed normality test (Shapiro-Wilk test). *p* values were shown and assessed by Mann-Whitney test **(A–D)**. All of the *p* values were labelled in the figure and *p* < 0.05 was considered to indicate statistical significance. AAV-D, AAV-5-gasdermin D; GSDMD, gasdermin D.

To further confirm the effects of gasdermin D suppression on atherosclerosis, AAV-5- gasdermin D (AAV-D) was administered to suppress gasdermin D expression in the arteries of ApoE^−/−^ mice who were fed a HFHC diet for 12 weeks. ORO staining revealed that the area of atherosclerotic lesions in the whole aortic artery was smaller in ApoE^−/−^ mice treated with AAV-D than in those in the AAV-control group (Figure 5A). Similarly, H&E and ORO staining showed that atherosclerotic lesions were less severe at the aortic root in the ApoE^−/−^ mice treated with AAV-D than in the mice in the AAV-control group (Figure 5B). Flow cytometry and immunofluorescence staining revealed that the fractions of F4/80+ macrophages in the whole aortic artery and aortic root were smaller in ApoE^−/−^ mice treated with AAV-D than in ApoE^−/−^ mice treated with AAV-control (Figures 5C,D). These preliminary data indicate that the suppression of gasdermin D attenuated atherosclerotic plaque progression and macrophage infiltration in ApoE^−/−^ mice.

## Discussion

This study revealed that caspase-11-gasdermin D-mediated pyroptosis and the subsequent proinflammatory response in macrophages participate in the pathogenesis of atherosclerosis. First, we found this potential mechanism by exploring transcriptomic data from hyperlipidemic mice available in the GEO database. Second, an *in vivo* study showed that caspase-11-gasdermin D activation occurred in response to HFHC diet consumption in ApoE^−/−^ mice, while the genetic deletion of caspase-11 or AAV-mediated suppression of gasdermin D largely attenuated the volume and macrophage infiltration of atherosclerotic lesions. Finally, an *in vitro* mechanistic study also supported the conclusion that lipid-associated inflammation occurs partly via caspase-11-gasdermin D-mediated pyroptosis in macrophages. Therefore, targeting caspase 11-gasdermin D could serve as an alternative strategy for the treatment of atherosclerosis.

Atherosclerosis is a disease with low-grade chronic inflammation that can be caused by lipids and other factors, including elevated blood pressure, tobacco use, metabolic disorder, and insulin resistance (Libby et al., 2019; Lu et al., 2020). The accumulation of lipids and proinflammatory cells in the arterial intima is regarded as the main cause of the initiation and progression of atherosclerosis (Back et al., 2019; Cai et al., 2020). Lipoproteins less than 70 nm in diameter can pass through the endothelial barrier and enter the arterial intima from the circulation, becoming retained lipoproteins through the interaction with intimal extracellular proteoglycans (Nordestgaard et al., 1995). Retained lipoproteins are susceptible to modification by intimal oxidizing agents, proteases, and lipases, which leads to the generation of lipid mediators that further increase inflammation and atherogenesis in the arterial intima (Houde and Van Eck, 2018). Canonical inflammasomes NLRP3 is activated by various endogenous danger signals, such as oxidized low-density lipoprotein and cholesterol crystals, and is abundantly presented in atherosclerotic lesions, which contributes to the vascular inflammatory response accelerating atherogenesis development and progression (Duewell et al., 2010; Zhang et al., 2018). The uptake of oxidized, proteolyzed or lipolyzed lipoproteins by macrophages and dendritic cells induces the activation of the canonical Nlrp3 inflammasome (Westerterp et al., 2017).

The recent discovered the non-canonical inflammasome Caspase-11 is a cytosolic LPS receptor that directly binds to lipid A on LPS and mediates cell pyroptosis (Deng et al., 2018; Wu et al., 2018). Human caspase-4/5 and mouse caspase-11 are homologues. Recent studies have revealed that ox-PAPC and stearoyl lysophosphatidylcholine have structural similarities with lipid A, which can bind to caspase-11 (Zanoni et al., 2016). As lipoproteins are cargo proteins for a large variety of fatty acids and cholesterol, we were the first to identify that lipid toxicity triggers noncanonical caspase-11 inflammasome activation in macrophages and promotes the progression of atherosclerosis. Given the evidence, activation of caspase-11 non-canonical inflammasomes by intracellular lipidprotein is distinct from canonical inflammasome activation and provides a new paradigm in macrophage-mediated inflammatory responses.

It has been proven that gasdermin D, a generic substrate for inflammatory caspases, such as caspase-1, caspase-4, caspase-5 and caspase-11, plays a specific role in inflammatory caspase-mediated pyroptosis and acts as a downstream effector of multiple inflammasomes (Shi et al., 2015). Mechanistically, we found that gasdermin D was downstream of caspase-11 activation and induced pyroptosis in lipid-loaded macrophages and that caspase-11 deficiency blocked the activation of gasdermin D in atherosclerosis. Previous studies reported that inflammasome activation and its triggered pyroptosis occurs in endothelial cells (Wu et al., 2018), macrophages (Zheng et al., 2011), and vascular smooth muscle cells (VSMCs) (Bauriedel et al., 1998; Cai et al., 2015) during the pathogenesis of numerous cardiovascular diseases (Pan et al., 2018; Li et al., 2019). Our novel findings indicated that ox-LDL induced caspase-11-gasdermin D-mediated macrophages pyroptosis and inflammation is a critical mechanism promoting atherosclerosis. This study was not able to conclude that pyroptosis in other cell types is also involved in the pathogenesis of atherosclerosis. Recent studies found that caspase-1 associated pyroptosis occurs in VSMCs and its activation potentiated the progression of atherosclerosis (Li et al., 2020). However, the role of caspase-11-gasdermin D-mediated pyroptosis of VSMCs and other cells in atherosclerosis warrant to be investigated in further studies.

Based on previous findings focusing on the mechanism of atherosclerosis, antilipoprotein therapies, anti-inflammatory therapies, and immunomodulatory therapies have been tested or are currently being tested in clinical trials (Cai et al., 2017; Zhao and Mallat, 2019). Antilipoprotein therapies are represented by statins, which target HMG-CoA reductase (Jain and Ridker, 2005). In addition, anti-proprotein convertase subtilisin/kexin type 9 (PCSK9) antibodies reduce plasma LDL-C levels by prolonging the lifespan of low-density lipoprotein receptor (LDLR) and inhibiting its degradation (Sabatine et al., 2017). Anti-inflammatory therapies include antibodies or inhibitors targeting ox-LDL, lipoprotein-associated phospholipase A2, secretory phospholipase A, 2/5-lipoxygenase, and 5-lipoxygenase activating protein (Hakonarson et al., 2005; Tardif et al., 2010; Nicholls et al., 2014; O'Donoghue et al., 2014; Lehrer-Graiwer et al., 2015). Inhibitors of the NLRP3 inflammasome and Il-1β pathway (colchicine, MCC950, anakinra, etc.) are the main drugs applied as immunomodulatory therapies (Fuster et al., 2017). Our discovery provides new perspectives on immunomodulatory therapies for vascular inflammation and atherosclerosis.

## Conclusion

Caspase-11-gasdermin D-mediated pyroptosis and the subsequent proinflammatory response in macrophages participate in the pathogenesis of atherosclerosis. Therefore, targeting the caspase 11-gasdermin D pathway may serve as an alternative strategy for the treatment of atherosclerosis.

## Data Availability

The datasets presented in this study can be found in online repositories. The names of the repository/repositories and accession number(s) can be found below: NCBI GEO; GSE171413.
