# Supplementary figures and images for: Caspase-11-Gasdermin D-Mediated Pyroptosis Is Involved in the Pathogenesis of Atherosclerosis
Source: Front Pharmacol. 2021 Apr 26;12:657486. doi: 10.3389/fphar.2021.657486 (PMC8109243; doi:10.3389/fphar.2021.657486)

Figure S1

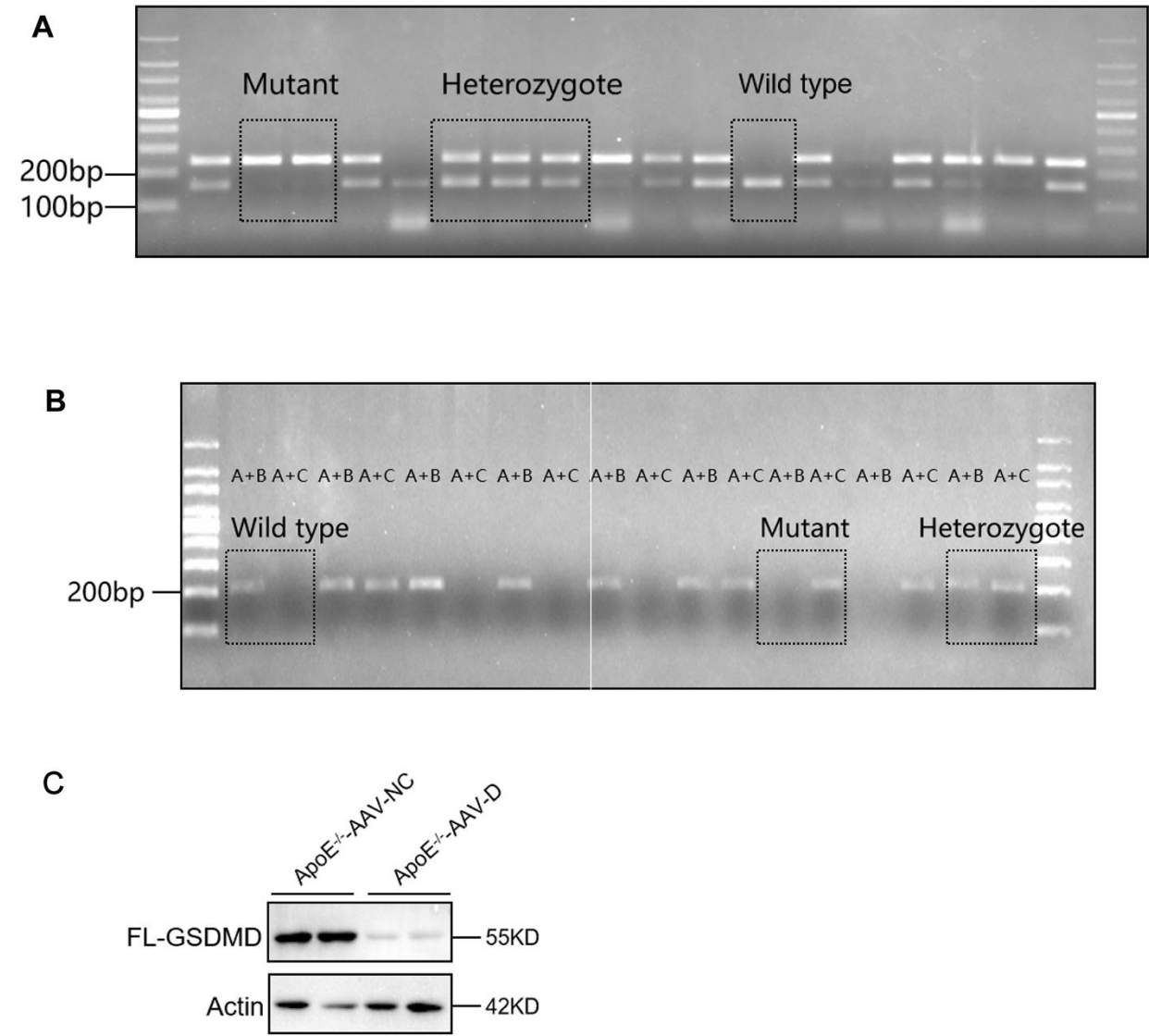

Figure S2

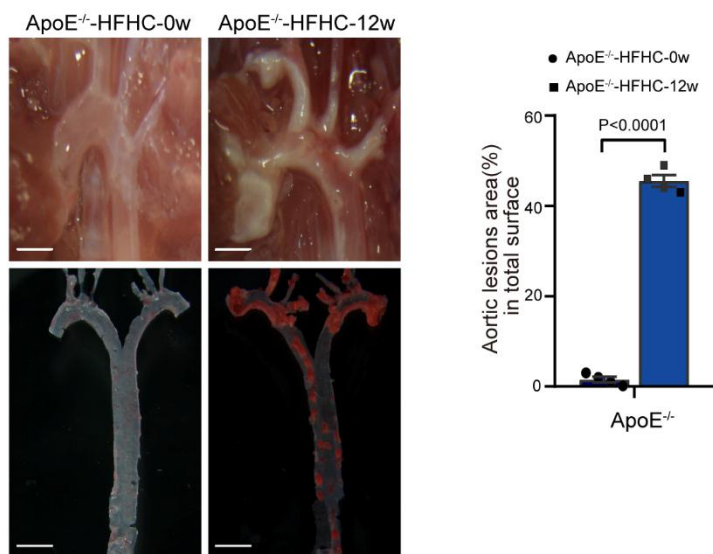

Figure S3

A

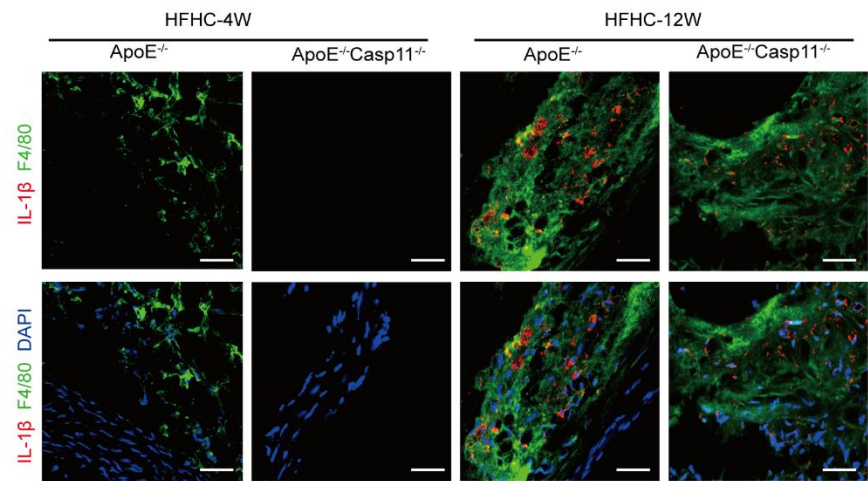

B

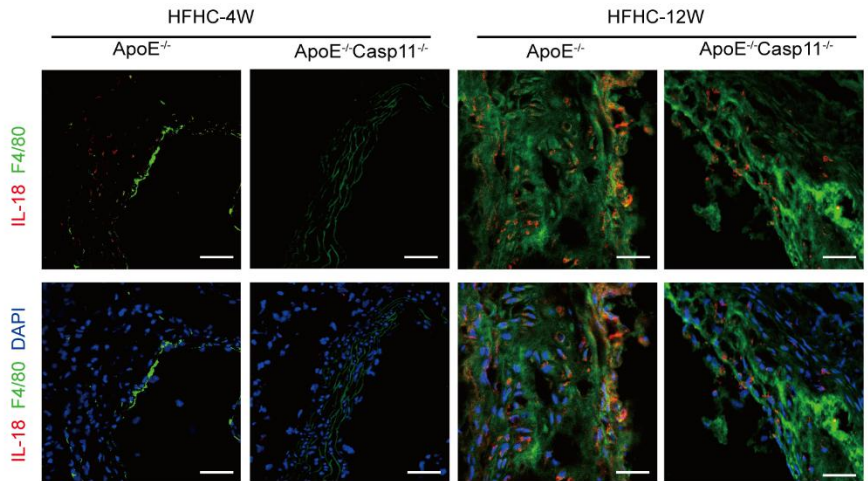

C

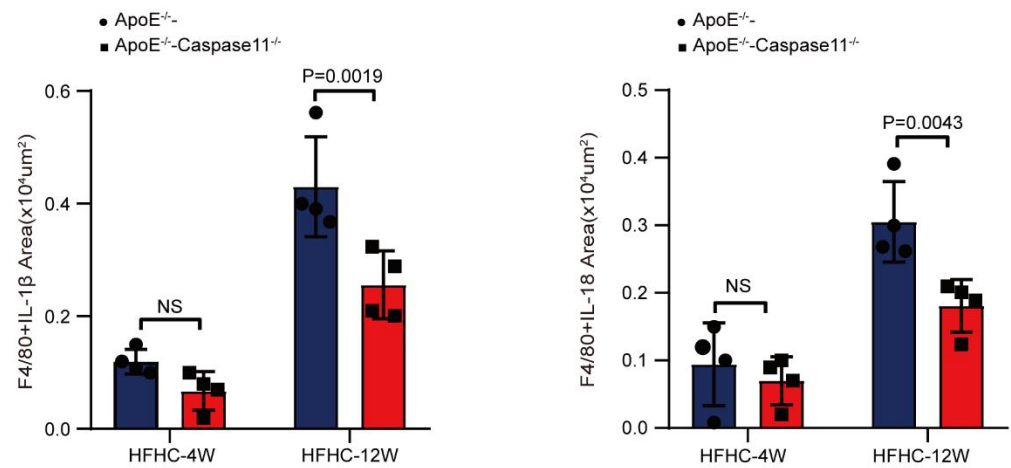

Figure S4

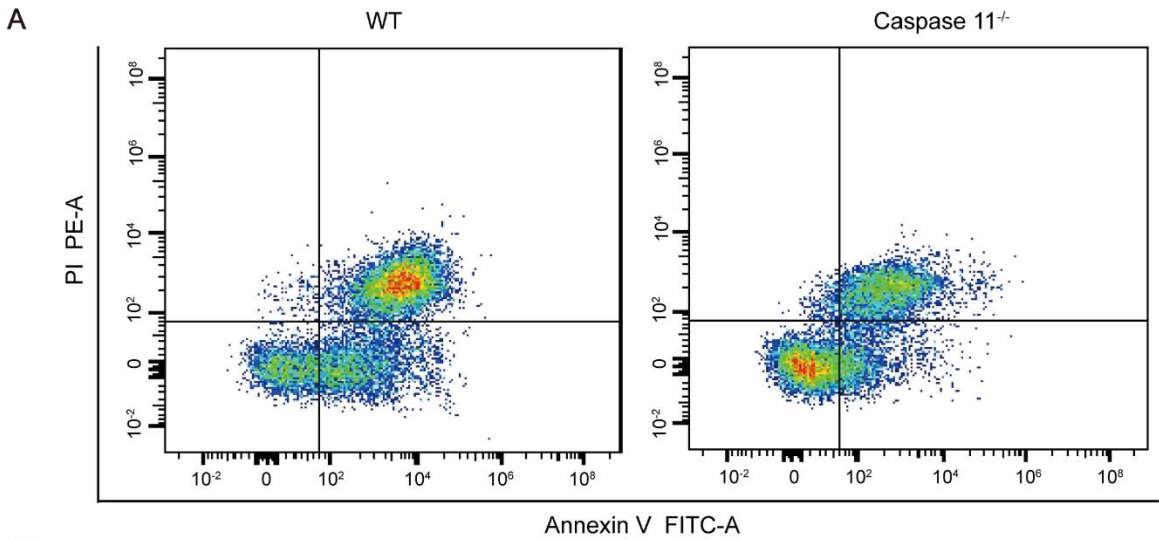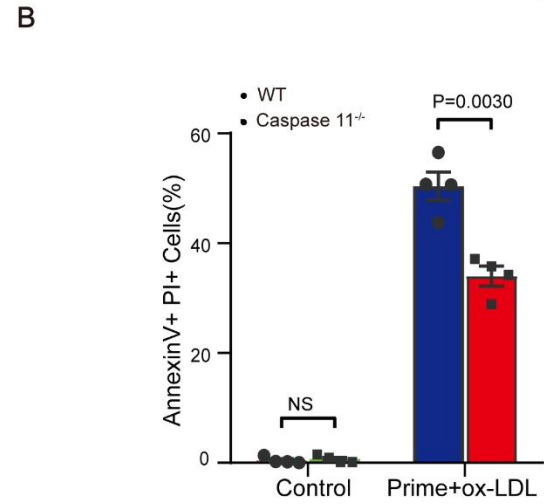

Supplement: Supplementary file 1 [file datasheet1.pdf]
